# Apelin: A Promising Therapeutic Target for Degenerative Diseases of the Musculoskeletal System

**DOI:** 10.14336/AD.2025.0555

**Published:** 2026-08-02

**Authors:** Jinkun Xu, Hengzhen Li, Jianfeng Sun, Gaoming Liu, Yuming Yao, Dongliang Yuan, Wenfeng Xiao, Yusheng Li

**Affiliations:** ^1^Deparment of Orthopedics, Xiangya Hospital, Central South University, Changsha 410008, Hunan, China.; ^2^Xiangya School of Medicine, Central South University, Changsha 410083, Hunan, China.; ^3^National Clinical Research Center for Geriatric Disorders, Xiangya Hospital, Central South University, Changsha 410008, Hunan, China.; ^4^Teaching and Research Section of Clinical Nursing, Xiangya Hospital of Central South University, Changsha, Hunan, China.

**Keywords:** Apelin, Apelin Receptor, Aging, Degenerative Diseases, Musculoskeletal System

## Abstract

Degenerative diseases of the musculoskeletal system, including sarcopenia, osteoarthritis, and osteoporosis, represent a cluster of age-associated conditions pathologically linked to chronic inflammation, oxidative stress imbalance, and endocrine dysregulation. These musculoskeletal diseases have emerged as a critical public health concern in aging populations worldwide, significantly compromising functional mobility and overall quality of life. Apelin, an endogenous ligand for the G protein-coupled receptor apelin receptor (APJ), constitutes a crucial component of the apelin/APJ signaling system that exhibits extensive distribution throughout human physiological systems. The multifaceted regulatory roles of apelin in cellular homeostasis, tissue remodeling, and inflammatory responses position it as a promising therapeutic target for these degenerative conditions. This review mainly discusses the effects of apelin on the occurrence and development of degenerative diseases in different musculoskeletal systems, aiming to establish a scientific foundation for future investigations and to identify novel therapeutic strategies for these conditions.

## 1. Introduction

The apelin system represents a fundamental physiological regulatory network that plays crucial roles in maintaining systemic homeostasis. It is composed of three essential components: apelin receptor (APJ), along with apelin and elabela, the two endogenous ligands [1]. Apelin, a multifunctional bioactive peptide, serves as the principal endogenous ligand for the APJ [2]. It is extensively distributed throughout the body, even in peripheral tissues like lungs, kidney, heart, bones, joints and muscles, and also the central nervous system [3-10]. Notably, adipocytes have been identified as primary synthesis sites for apelin, establishing its dual classification as both a neuropeptide and adipocytokine [11]. The past decade has witnessed exponential growth in apelin-APJ system research, driven by emerging insights into its multifaceted regulatory capacities spanning energy metabolism, inflammatory modulation, and tissue homeostasis maintenance.

Degenerative diseases of the musculoskeletal system are a group of diseases related to aging, inflammation, oxidative stress, and hormonal changes, including but not limited to sarcopenia, osteoarthritis, osteoporosis, and intervertebral disc degeneration [12, 13]. With the accelerating global aging trend, degenerative musculoskeletal disorders have emerged as a pressing public health crisis [14]. Despite decades of mechanistic investigations, the etiological complexities underlying these age-related pathologies remain elusive. Apelin/APJ system greatly impacts physiological processes and diseases of musculoskeletal system [15],demonstrating an apparent duality in musculoskeletal homeostasis. Activation of the apelin/APJ system can promote bone formation [16]. It can also attenuate osteoporosis [17] while induce osteoarthritis (OA) [18]. Some scholars have found that apelin can promote skeletal muscle formation [19] and plays an positive role in accelerating metabolism of the skeletal muscle [20].

## 2. Apelin/APJ System

### 2.1 Structure of Apelin/APJ

APJ, classified as a G protein-coupled receptor (GPCR) family member, was separated in 1993 [21]. Structural analysis reveals APJ contains 380 amino acid residues, featuring the characteristic seven-transmembrane hydrophobic domains of GPCRs, along with conserved phosphorylation sites for cAMP-dependent protein kinase, in addition to palmitoylation and glycosylation sites [22]. APJ receptor protein is closely related to angiotensin II receptor type 1 (AT1R) [23]. First discovered in 1998, apelin is an endogenous APJ-binding peptide hormone purified from bovine stomach tissue [24]. Initially translated as a 77-residue precursor peptide, apelin is post-translationally cleaved to produce its functional variants [25]. This pre-peptide is composed of 77 amino acid residues, including a putative N-terminal secretory signal peptide of 22 amino acids and a C-terminal domain of 55 amino acids containing a receptor binding site [26]. The C-terminal region of apelin contains the critical binding determinants for interaction with APJ [27].

To comprehensively understand the biological functions of apelin, it is essential to conduct in-depth exploration of its diverse subtypes. These subtypes, although originating from the same gene, exhibit significant structural differences due to different splicing modes. Next, we will provide a detailed analysis of the molecular characteristics and unique structural properties of each subtype of apelin.

### 2.2 Subtypes of Apelin

Apelin is considered to belong to the adipokine family and is mainly encoded by the apelin gene (*APLN*). In humans, the *APLN* resides on the long arm of chromosome X (Xq25-26.1), encoding a 77 amino acid proprotein [21]. This propeptide contains a site with many basic amino acid residues (such as Arg-Arg and Arg-Lys), which is cut into different subtypes by the endoprotease. For instance, dibasic residues contained within the motif RR-XX-RR are recognized and cleaved by the proprotein convertase subtilisin/kexin type 3 (PCSK3), commonly known as furin. Thus forms a biologically active apelin fragment family, including but not limited to apelin-13, apelin-17 and apelin-36 [21, 28, 29]. The C-terminus of all apelin subtypes has a common 12-amino acid residue sequence structure (Arg-Pro-Arg-Leu-Ser-His-Lys-Gly-Pro-Met-Pro-Phe) which is exhibits remarkable phylogenetic conservation across diverse species. And it is essential for receptor activation [30]. Angiotensin-converting enzyme 2 (ACE2), a circulating metalloprotease, post-translationally processes apelin by cleaving the C-terminal phenylalanine residue from all its isoforms [31]. The removal of phenylalanine is related to the decrease of cell reaction [32].

**Figure 1.**
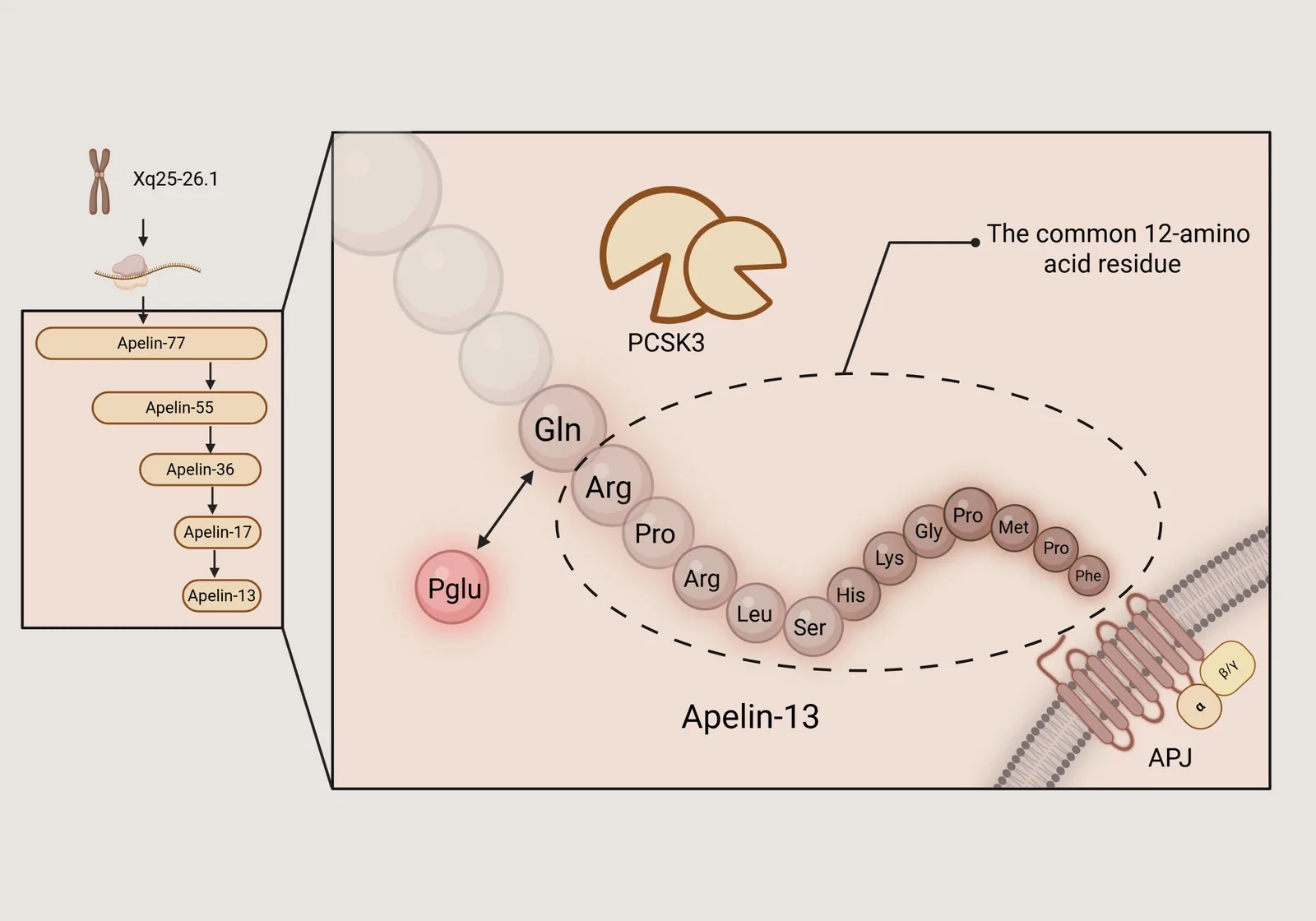
***APLN* is located on the human chromosome Xq25-26.1, encoding a 77 amino acid proprotein (apelin-77)**. After cut into different subtypes by the endoprotease (such as PCSK3), it gradually becomes a shorter subtype. The C-terminus of all apelin subtypes has a common 12-amino acid residue sequence structure, which has the ability to specifically bind to APJ. The shorter the length, the stronger the subtype activity. Apelin-13 and its pyroglutamic acid modified form Pyr-Apelin-13 are the most active subtypes. Created in BioRender.

The main difference between different subtypes is the size of peptide. The 77-amino acid precursor undergoes sequential proteolytic cleavage, initially yielding apelin-55, which is subsequently processed into shorter active forms including apelin-36, apelin-17, and apelin-13 [33]. Different apelin subtypes have different receptor binding affinity and lead to different APJ intracellular transport. Notably, this ability seems to be size-dependent [24]. Shorter apelin subtypes like apelin-13 and apelin-17 seem to be more effective APJ activators than apelin-36 [34]. Comparative functional analyses reveal apelin-13 and its pyroglutamate-modified derivative (Pyr-apelin-13) exhibit the highest biological activity among all apelin isoforms [35]. Apelin-13 is conserved in various species [36], and it can be detected in human plasma [37]. Structural characterization reveals the N-terminal RPRL motif of apelin-13 adopts a classical β-turn conformation, as demonstrated by nuclear magnetic resonance spectroscopy [38], which is related to the specific recognition function of the receptor [39]. Therefore, shorter isoforms such as apelin-9, apelin-10 and apelin-11 have also been shown to be inactive [34, 40]. And it was finally found that apelin could not be truncated to a shorter form than apelin-12 at the N-terminus, otherwise it would lose the ability to bind to ARJ and correspondingly lose signal transduction activity [27]. Pyr-apelin-13 is generated through post-translational cyclization of apelin-13's N-terminal glutamine into pyroglutamic acid, a modification that confers resistance to N-terminal degradation and enhances plasma stability [41]. It has been reported that apelin-17 has a better performance in recruiting β-arrestin [42], and inhibited intracellular cAMP production [43]. Apelin-36 can activate protein kinase C, promote cell proliferation and inhibit apoptosis [44-46]. Following apelin-36-induced internalization, the APJ receptor undergoes β-arrestin1-mediated trafficking to endolysosomal compartments via a Rab7-regulated pathway [47]. Prior to 2014, apelin was thought to be the exclusive endogenous APJ agonist, when research revealed Elabela as a second physiologically relevant ligand [48] (Fig. 1).

The various subtypes of apelin not only have different structures, but also have different biological functions, mainly due to the different signaling pathways activated by their binding to receptors, thus exerting diverse physiological effects. Next, we will explore the key signaling transduction mechanisms mediated by apelin.

### 2.3 Signal Transduction and Function of Apelin

The apelin/APJ system can be activated by different factors to initiate a series of subsequent signal transduction. One of these factors is hypoxia. Cellular responses to low oxygen are orchestrated by hypoxia inducible factor (HIF), a functional heterodimer formed by the interaction between HIF-1α and HIF-1β proteins [49]. HIF-1α has biological activity and functions as an essential mediator in oxygen homeostasis [50]. Research demonstrates that HIF-1α-mediated transcriptional activation upregulates both apelin and APJ expression under hypoxic conditions [51]. This has also been confirmed in bone marrow-derived mesenchymal stem cells (BMSC) [52]. In addition, bone morphogenetic protein (BMP) inhibited hypoxia-induced endothelial apelin upregulation [53]. Another study demonstrates that exposure to chemical hypoxia can also significantly increase the level of apelin in rat liver [54].

In addition to hypoxia, inflammation is another powerful factor in activating the apelin/APJ system [55]. Inflammation represents a protective physiological response triggered by pathogenic invasion or cellular injury [56]. The mediators involved in inflammation include, but are not limited to, inflammatory cytokines, chemokines, and proteolytic enzymes. The inflammatory cytokine network encompasses tumor necrosis factor-alpha (TNF-α), interleukin-1(IL-1), and interleukin-6(IL-6), which are produced by numerous cell types. As key effector cells, macrophages and mast cells orchestrate multiple inflammatory processes through endothelial / leukocyte activation and acute-phase protein induction [57]. Inflammatory response can lead to the activation and conduction of apelin signal in vivo. For instance, inflammatory cytokines like IL-6 and interferon-gamma (IFN-γ) can increase intestinal apelin levels by activating Jak/Stat3 signal transduction in vivo and in vitro [58]. Another animal experiment demonstrated that pancreatitis could activate apelin, and enhanced apelin-APJ signaling regulates pancreatitis and fibrosis during pancreatitis [59].

Functioning as an adipokine, apelin demonstrates significant involvement in obesity-related metabolic dysregulation, including the development of insulin resistance [60, 61]. Prolonged obesity and excessive nutrient intake promote insulin resistance and sustain low-grade inflammation [62]. Obesity and insulin increase the content of apelin in adipocytes. A study has shown that plasma insulin directly promotes apelin production and gene transcription in adipocytes by activating PI3K, ERK1/2 and PKC in vitro and in vivo [60]. Comparative analyses reveal significantly higher plasma apelin concentrations in both insulin-resistant subjects and severely obese type 2 diabetics versus healthy control populations [63-65]. Relevant evidence suggests that targeting apelin/APJ signal transduction may represent a potential new way to design insulin resistance therapy [66].

In clinical trials, exercise has also been shown to be associated with apelin. Long-term exercise can increase the content of apelin in obese individuals, thus delaying the progression of metabolic diseases [67]. In turn, apelin can be used as an exercise-sensitive factor to increase the potential for exercise [68]. While another clinical trial showed that exercise seemed to have no effect on apelin levels in individuals with type 2 diabetes [69]. This may be due to the heterogeneity of different diseases.

As a GPCR, APJ exhibits significant heterogeneity in signal transduction. This receptor mainly works by coupling with the Gα_i/o_ protein subtype, where the Gαi protein subunit mediates adenylate cyclase inhibition, leading to decreased intracellular cAMP production, and thereby affect the activation of protein kinase A (PKA) [70, 71] . In different cell lines, APJ exhibits a clear preference for G protein conjugation: it preferentially binds to Gα_i1/2_ in CHO and HEK293 cells, while it tends to bind to Gα_i3_ in HUVEC cells [72-74].

The C-terminal domain of APJ undergoes phosphorylation at five distinct serine residues (Ser335, Ser339, Ser345, Ser348, and Ser369), a critical modification required for β-arrestin recruitment [75, 76]. APJ mutation does not interfere with the phosphorylation process of β-arrestin itself, but significantly changes its signal transduction function, especially the role of Ser335 and Ser339 sites. Briefly, Ser335 mutation leads to a decrease in the binding ability of the receptor to β-arrestin1 / 2 and AP2.Ser339 mutation completely blocks the interaction between the receptor and GRK2 / β-arrestin1 / 2 under apelin-36 stimulation, which leads to the failure of normal internalization of the receptor, thus interrupting the β-arrestin-dependent ERK1 / 2 activation pathway.

In the pathological state of ischemia or hypoxia, apelin exerts a significant protective effect by activating the reperfusion injury salvage kinase (RISK) signaling pathway [77]. This protective mechanism involves three key signaling molecules in the RISK pathway: PI3K/Akt pathway, p44/42 MAPK and ERK1/2. Animal experiments further confirmed that apelin can specifically activate the two important signal transduction pathways of PI3K / Akt and p44 / 42 MAPK under ischemia and hypoxia conditions [36]. Thus, effectively counteract the cell damage caused by ischemia and hypoxia by promoting the molecular mechanism of cell growth and survival. Apelin plays a synergistic regulatory role of multiple targets to achieve the protective effect on cells.

**Figure 2.**
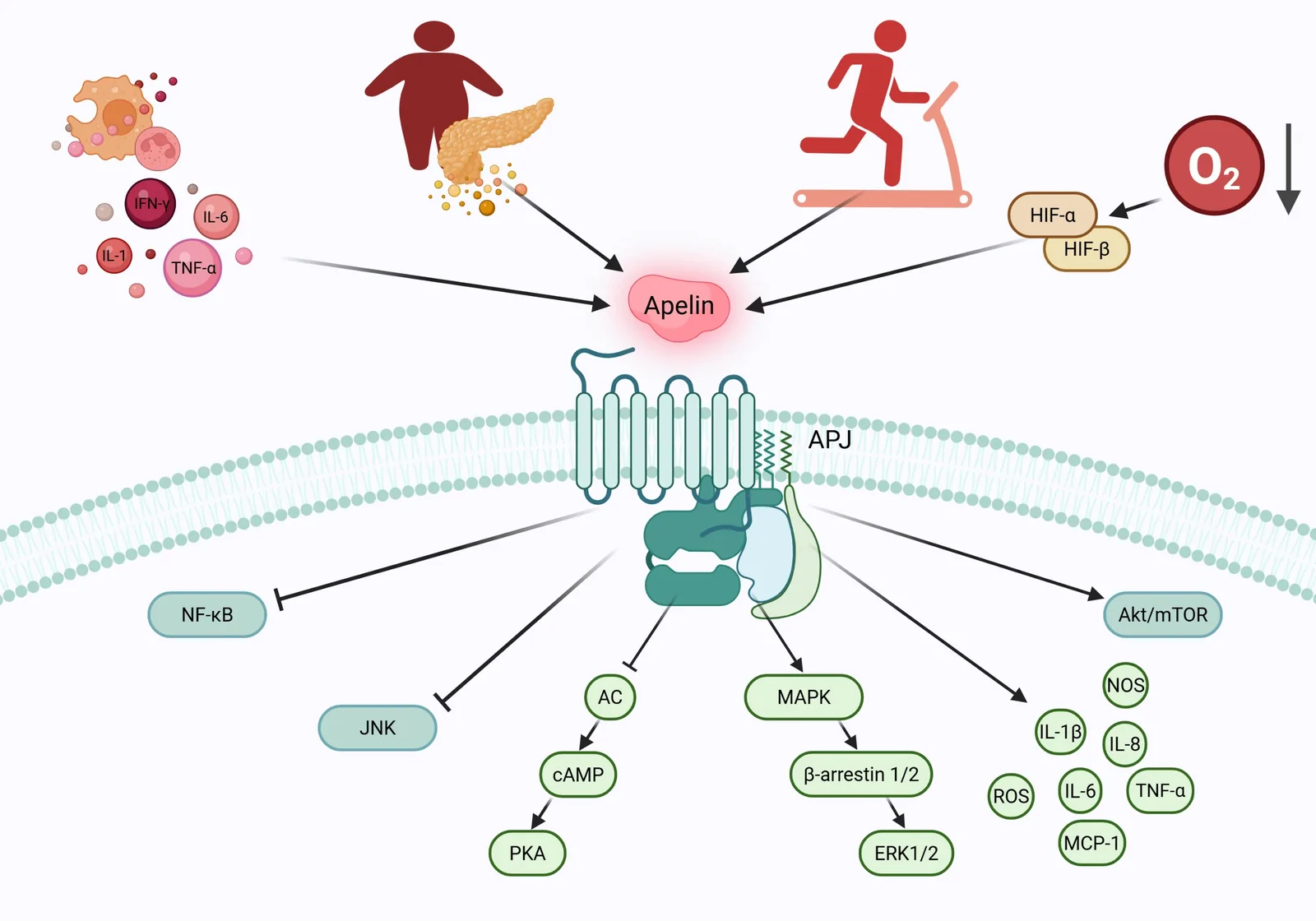
**Many factors can activate the Apelin/APJ system, such as inflammation, obesity and insulin resistance, exercise, and the production of HIF under hypoxic conditions**. Apelin binds to APJ and conducts downstream signal transduction through G protein. Such as inhibiting NF-κB and JNK signaling pathways, down-regulating the content of PKA. It can also activate MAPK/β-arrsetin / ERK cascade signaling, promote cytokine expression, activate Akt / mTOR signaling pathway, etc. Created in BioRender.

The apelin/APJ system exerts potent anti-inflammatory actions by modulating key signaling pathways - suppressing NF-κB and JNK activation while inhibiting pro-inflammatory mediators including TNF-α, IL-1β/6/8, monocyte chemoattractant protein-1 (MCP-1), nitricoxidesynthase (NOS), and reactive oxygen species (ROS) production [22]. Research has shown that apelin exerts specific anti-inflammatory mechanisms in different organ systems. In a rat model of heart failure, apelin-13 significantly inhibits ROS generation and NF-κB phosphorylation by promoting the Akt/eNOS phosphorylation pathway, thereby downregulating the expression of pro-inflammatory factors such as TNF-α, MCP-1, and IL-1β, effectively improving vascular lesions and inflammatory responses [78]. In addition, multiple studies have confirmed that exogenous apelin treatment can significantly reduce lung tissue inflammation infiltration and pathological damage by inducing activation of the PI3K/Akt signaling pathway [79]. In the mouse model of diabetes nephropathy, exogenous apelin-13 treatment can up regulate the expression of APJ receptor and catalase, reduce the structural changes of kidney caused by diabetes, and reduce the expression of renal inflammatory mediators such as MCP-1, vascular cell adhesion molecule-1(VCAM-1) by inhibiting NF-κB signaling pathway [80]. In the intestinal system, the apelin/APJ system promotes lymphatic drainage by synergistically activating the Akt and ERK1/2 signaling pathways, thereby reducing the infiltration of colonic mucosal inflammatory cells [81]. These findings collectively reveal the molecular basis of apelin's multi organ protective effects under different pathological conditions. Studies indicate that the biological actions of administered apelin are context-specific, varying significantly with dosage and delivery method [82] (Fig. 2).

Given the important role of the apelin signaling pathway in cellular regulation, its association with degenerative diseases of the musculoskeletal system has received increasing attention in recent years. Research has shown that abnormal expression of the apelin/APJ system may be involved in the pathological processes of various degenerative diseases. Next, we will focus on exploring the potential mechanisms of apelin in degenerative diseases of the musculoskeletal system such as sarcopenia, osteoarthritis, and osteoporosis.

## 3. Apelin and Degenerative Diseases of the Motor System

### 3.1 Apelin and Sarcopenia

Sarcopenia represents a progressive, systemic musculoskeletal disorder characterized by accelerated decline in both muscle mass and strength, clinically associated with elevated risks of falls, functional impairment, frailty, and mortality [83].

Sarcopenia pathogenesis is multifactorial, with current research highlighting several key contributors: age-related physiological decline, hormonal dysregulation, chronic low-grade inflammation, oxidative stress imbalance, α-motor neurons degeneration, mitochondrial dysfunction, dysregulated myocyte autophagy, accelerated apoptosis of myonuclei, and satellite cell dysfunction [84]. Mitochondrial dysfunction has emerged as the central pathophysiological mechanism of skeletal muscle aging and sarcopenia development [85], primarily attributable to the tissue's high oxidative metabolic demands that predispose myocytes to ROS-mediated damage when mitochondrial integrity is compromised [86].

Emerging evidence demonstrates an inverse correlation between apelin levels and sarcopenia prevalence [87, 88]. Apelin can affect the progression of sarcopenia through a variety of mechanisms. Apelin exerts multifactorial therapeutic effects through coordinated activation of AMPK/AKT/P70S6K signaling axes, which promote mitochondrial biogenesis and stimulate muscle protein synthesis [89]. These combined mechanisms ultimately preserve metabolic homeostasis and rejuvenate satellite cell functionality in aging musculoskeletal systems [90]. Local apelin secreted by muscles increases their endurance by increasing mitochondrial number and activating AMPK, which indicates that there may be an autocrine regulatory loop in skeletal muscle [91]. Apelin stimulates epigenetic remodeling of the peroxisome proliferator-activated receptor gamma coactivator-1α promoter through DNA demethylation, upregulating its expression in developing muscle tissue and promoting mitochondrial biogenesis [92].The level of apelin is positively correlated with the number of capillaries, which increases the angiogenesis of skeletal muscle and plays an active role in muscle physiological changes [93]. In addition to its other roles, apelin enhances cellular autophagy, reduces muscular inflammation, and facilitates the regenerative capacity of satellite cells [94]. Apelin as a biomarker and a component of the diagnostic criteria for frailty shows a statistically significant correlation [95]. In many animal experiments also confirmed the above conclusion [87, 90, 96-100] that apelin helps to fight the progress of sarcopenia. A clinical trial has also shown that muscle strength after resistance training in middle-aged people is associated with muscle mass and transcriptional adaptation of apelin signaling [101].

A variety of mechanisms are also involved in the regulation of apelin content. At the micromolecular level, some cytokines have been shown to regulate the content of apelin. For example, the concentration of TGF-β increases with age, and high concentrations of TGF-β can reduce the expression of apelin, resulting in rotator cuff degeneration [102]. Another cytokine is TNF-α,which has been proved to be able to stimulate the expression of apelin in mouse and human adipose tissue [103, 104]. Interestingly, TNF-α can induce Apelin mRNA expression in muscle cells from young mice. However, TNF-a failed to induce apelin expression in aged mouse-derived muscle cells [103]. The specific mechanism of TNF regulating apelin needs to be further explored. In the macro level, it has been reported that age is one of the regulatory factors, and the level of apelin in humans and rodents is reduced in an age-dependent manner [105]. Another well-known macro-regulatory factor is exercise. After a period of aerobic exercise, the expression of apelin in male or female subjects was upregulated [96, 106, 107]. At the same time, studies have shown that after an acute resistance training, the release of apelin in athletes is also more than that in non-athletes [108].

So apelin 's effect against sarcopenia seems certain, but interestingly, some literature supports different results. A study has shown that apelin is significantly increased in patients with sarcopenia compared to non-sarcopenia subjects [109]. Contradicting these findings, certain cross-sectional analyses have failed to establish a significant correlation between circulating apelin concentrations and sarcopenia prevalence in elderly populations [110, 111]. This may be due to the fact that sarcopenia involves a series of pathophysiological processes, such as hypoxia and inflammation. As mentioned earlier, hypoxia and inflammation can activate the apelin/APJ system, and apelin can exert hypoxia protective and anti-inflammatory effects through different mechanisms. Therefore, at a certain stage of disease progression, due to the compensatory effect of the body, the content of apelin in patients with sarcopenia may increase. Apelin is related to aging in the body, and it decreases with age [19]. Therefore, apelin in elderly patients with sarcopenia is subjected to two completely opposite driving forces, resulting in a statistically "unrelated" result. So, it is necessary to conduct larger scale clinical trials involving different populations to further explore the impact of apelin on sarcopenia.

### 3.2 Apelin and Osteoarthritis

Osteoarthritis (OA) represents the predominant arthritic disorder in clinical populations [112]. As a chronic joint pathology, OA manifests through hyaline cartilage erosion, inflammatory synovitis, aberrant bone remodeling beneath the cartilage, and bony outgrowth formation [113]. The hallmark pathological feature of OA is progressive deterioration of articular cartilage [114]. Chondrocyte activation in OA drives the release of catabolic factors such as MMP-1/3/13 and ADAMTS-4/5, contributing significantly to joint destruction [115].

Many studies have pointed out that apelin is a risk factor for OA. Apelin has been proved to promote cartilage catabolism by many studies [116-118]. Investigations have demonstrated elevated apelin levels in the synovial fluid of knee OA patients compared to healthy controls [117, 119]. Reducing the expression of apelin can reduce the severity of OA cartilage and may become one of the targets for future OA treatment [120]. In view of these findings, apelin can be considered as a risk factor for OA pathophysiology [121]. Apelin has been used as a biomarker to evaluate the efficacy of OA patients in clinical trials [122].

Apelin promotes the progress of OA through a variety of mechanisms, among which the relationship between apelin and VEGF and hydrolase is deeply studied. VEGF is a powerful angiogenic stimulator that contributes to the inflammatory process [123]. The level of apelin in the synovial fluid of the temporomandibular joint is positively correlated with the patient 's age and VEGF level [119]. Comparative analyses reveal markedly elevated VEGF expression in OA patients relative to healthy controls [124, 125]. At the genetic level. Genetic variants in VEGF are strongly associated with elevated risk of osteoarthritis development [126]. A randomized controlled trial demonstrated that elevated VEGF concentrations in both plasma and synovial fluid correlate strongly with radiographic disease severity in knee OA [127]. Apelin can also inhibit the expression of miR-150-5p through the FAK / Src / Akt signaling cascade, thereby enhancing VEGF expression and angiogenesis and promoting the progression of OA [128].

As mentioned above, although OA is a disease involving the entire joint, it is characterized by progressive decomposition and loss of articular cartilage [129]. Many hydrolases can promote the decomposition of cartilage and extracellular matrix (ECM). Among them, matrix metalloproteinase (MMP) is considered to be the central mediator of cartilage destruction networks in OA pathogenesis [130]. Apelin promotes chondrocyte proliferation while upregulating expression of catabolic factors (MMP-1, -3, -9 and IL-1β) and downregulating collagen II production. Notably, IL-1β serves as a potent cartilage-degrading cytokine [131]. In synovial cells, the expression of IL-1β is inhibited by miR-144-3p, and the apelin/APJ system can inhibit the expression of miR-144-3p by phosphorylating PI3K and extracellular signal-regulated kinase (ERK), thereby up-regulating IL-1β and inducing OA [120]. As crucial mediators of cartilage degeneration, ADAMTS enzymes specifically target and degrade chondrocyte extracellular matrix proteins, resulting in structural deterioration [132]. Through apelin treatment, it was observed that the mRNA levels of ADAMTS-4 and -5 were significantly increased, and histological evaluation revealed the depletion of proteoglycans in articular cartilage [116]. These findings suggest that apelin promotes cartilage catabolism and is a risk factor for OA pathophysiology.

One of the clinical manifestations of osteoarthritis is joint pain, and apelin can also cause joint pain through different mechanisms. KEGG pathway analysis of upregulated differentially expressed genes in CD39+CD55- fibroblasts revealed significant enrichment in both the Apelin signaling pathway and cGMP-PKG cascade, potentially implicating these mechanisms in pain pathogenesis [133]. Emerging evidence suggests synovial-derived VEGF contributes to OA-associated nociception, potentially through functional interactions with apelin signaling cascades [123].

### 3.3 Apelin and Osteoporosis

Osteoporosis represents a systemic skeletal disorder marked by diminished bone density and deteriorated microarchitecture, leading to reduced mechanical integrity and heightened susceptibility to fractures [134]. Osteoporosis pathogenesis involves multifactorial mechanisms such as aging, diminished mechanical loading, endocrine dysfunction, and stress-induced alterations in osteogenic transcription, collectively disrupting the equilibrium between osteoclastic bone resorption and osteoblastic formation [135], that is, osteoclasts absorb bone through acidification and proteolysis, and osteoblasts secrete organic matrix into the absorption chamber [136].This imbalance can lead to osteoporosis. The bone marrow niche comprises a sophisticated cellular network that cooperates with BMSCs to sustain skeletal homeostasis through lifelong remodeling [137]. Apelin can affect the physiological changes of BMSCs, osteoblasts, and osteoclasts, thereby exerting a role in resisting osteoporosis.

BMSCs exhibit numerous advantageous properties that make them ideal candidates for regenerative medicine applications [138]. In BMSCs, the Wnt/β-catenin cascade serves as the primary mechanistic pathway through which apelin promotes osteogenesis. Dysregulation of this signaling axis represents a fundamental contributor to impaired bone formation in osteoporosis [139]. The apelin/APJ axis enhances BMSC osteogenesis by activating Wnt/β-catenin signaling, thereby upregulating the expression of bone-related genes and proteins [16]. For instance, procollagen type I propeptide (PINP) is a protein derived from osteoblasts and can be used as a biomarker to evaluate the efficacy of osteoporosis [140]. The carboxy-terminal telopeptide of type I collagen (ICTP) serves as a unique biochemical marker of bone resorption, representing a specific degradation product of type I collagen metabolism [141]. A direct association between apelin levels and PINP concentration, while demonstrating an inverse relationship with ICTP [17]. Lack of apelin can also lead to decreased expression of lysyl oxidase subtypes loxl3 and loxl4 [142]. Loxl can mediate the formation of collagen fibers, and collagen fibers can act as a three-dimensional scaffold for tissue mineral deposition, which is conducive to bone formation. [143]. In other aspects, apelin-13 activates mitophagy in BMSC and improves oxidative stress, thereby restoring osteogenic function through AMPK-α phosphorylation [144]. Preclinical studies demonstrate apelin's regulatory capacity in bone homeostasis, with apelin-13 exhibiting osteoprotective effects through modulation of BMSC inflammatory responses and apoptosis pathways [145].

Apelin also exerts its physiological effects by acting on osteoblasts and osteoclasts. Apelin/APJ receptor engagement triggers both PI3K/Akt pathway initiation and enhanced JNK phospho-activation, collectively driving osteoblast multiplication [146]. While for osteoclasts, apelin-13 inhibits osteoclastogenesis and bone resorptive activity by attenuating NLRP3 inflammasome-mediated pyroptosis through Nrf2 pathway activation [147]. From another perspective, relevant studies have shown that ROS accumulation can upregulate RANKL/M-CSF, activate MAPK and NF - κ B pathways, promote osteoclast differentiation, and exacerbate osteoporosis [148]. The activation of APJ can enhance mitochondrial autophagy, reduce ROS, and inhibit NLRP3/IL-1 β inflammatory response through the AMPK-BNIP3-PINK1-PARKIN axis [149]. Through this mechanism, the apelin/APJ system can indirectly inhibit the function of osteoclasts, thereby delaying osteoporosis.

**Figure 3.**
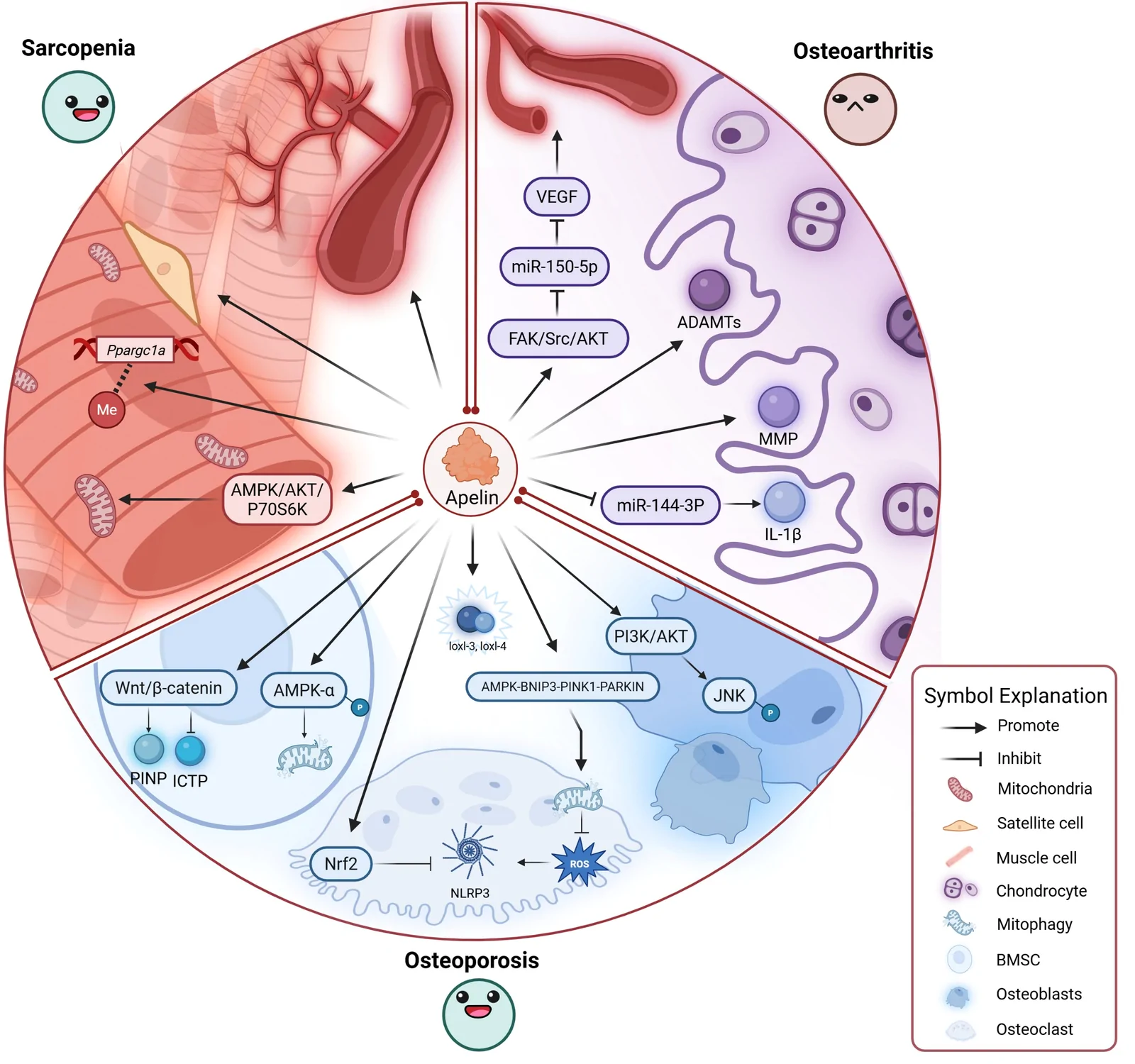
**Apelin affects degenerative diseases of the musculoskeletal system through different mechanisms**. Upper-left section: Apelin delays the progression of sarcopenia at the overall level. Apelin promotes angiogenesis The level of apelin is positively correlated with the number of capillaries, which increases the angiogenesis of skeletal muscle and plays an active role in muscle physiological changes. It rejuvenates satellite cell functionality in aging musculoskeletal systems. Apelin also induces DNA demethylation of the peroxisome proliferator-activated receptor gamma coactivator-1α (Ppargc1a) promoter and enhances its expression in fetal muscle and mitochondrial biogenesis. Apelin coordinated activation of AMPK/AKT/P70S6K signaling axes, which promote mitochondrial biogenesis and stimulate muscle protein synthesis. Upper-right section: Apelin promotes the pathophysiological progress of osteoarthritis. Apelin can inhibit the expression of miR-150-5p through the FAK / Src / Akt signaling cascade, thereby enhancing VEGF expression and angiogenesis and promoting the progression of OA. Apelin can induce the expression of a variety of hydrolases, such as ADAMTS, MMP, IL-1β. Thus, destroying the cartilage matrix and aggravating the severity of osteoarthritis. Lower-middle section: Apelin can fight the disease progression of osteoporosis. Apelin enhances BMSC osteogenic differentiation via Wnt/β-catenin signaling, upregulating bone-specific genes/proteins while concurrently activating AMPK-α-dependent mitophagy to alleviate oxidative stress and restore osteogenic capacity. Conversely, apelin deficiency reduces LOXL3/4 expression—enzymes critical for collagen fiber crosslinking, which provides the structural scaffold for mineral deposition during bone formation. Apelin can activate PI3K / Akt signal transduction and enhance JNK phosphorylation, thereby promoting osteoblast proliferation. Apelin-13 mitigates osteoclast formation and bone resorption by suppressing NLRP3 inflammasome-driven pyroptosis via activation of the Nrf2 signaling pathway. The activation of APJ can enhance mitochondrial autophagy, reduce ROS, and inhibit NLRP3/IL-1 β inflammatory response through the AMPK-BNIP3-PINK1-PARKIN axis. Created in BioRender.

At the genetic level, there are also valuable discoveries. [149]. Briefly, a study investigating ketogenic diet-induced osteoporosis revealed that KEGG pathway analysis highlighted significant enrichment of the apelin signaling pathway, PI3K-Akt signaling pathway and ECM-receptor interaction pathway, with these pathways exhibiting both high enrichment scores and a substantial number of differentially expressed genes. This indicates that genetic factors are also worth further investigation.

Apelin can delay the progression of osteoporosis [150, 151]. Nevertheless, apelin from different sources seems to have inconsistent effects on bone formation. Apelin can promote osteoblast proliferation and inhibit osteoblast apoptosis through the APJ / PI3 kinase / Akt pathway. Notably, apelin secreted by osteoblasts shows no significant influence on Runx2 levels, ALP enzymatic activity, or the production of osteocalcin and collagen I, suggesting it does not regulate osteogenic differentiation [146, 152, 153]. While muscle-derived apelin plays an active role in bone formation [105]. Interestingly, mice lacking adipose-derived apelin showed an increase in bone mass [154]. This also indicates that the musculoskeletal system is an interdependent organic whole, and the specific mechanism by which apelin regulates bone formation needs further exploration. Apelin has a variety of active forms, among which apelin-13 has important physiological significance [155]. It has been reported that apelin-13 levels are lower in osteoporosis patients than in non-osteoporosis individuals [17, 142]. The intrinsic mechanism of Apelin-13 regulation has potential therapeutic value in the treatment of osteoporosis.

Although apelin is associated with osteoporosis, a previous clinical study has shown that apelin is not an important independent predictor of bone strength [156]. Interestingly, previous studies have found that apelin knockout mice have increased bone mass compared with wild-type mice, indicating that apelin has an anti-synthetic metabolic effect in vivo [154]. Despite this, most current studies support apelin as a protective factor for osteoporosis. In order to fully tap the potential of Apelin in the field of osteoporosis treatment, the effect of Apelin on osteoporosis still needs further study (Fig. 3).

## 4. Discussion

Degenerative diseases of the motor system are a group of diseases related to multiple factors, which seriously affect the patient 's ability to work and lead to adverse sequelae such as fractures and even death [157]. Global population aging has precipitated a marked increase in the incidence and prevalence of locomotor system degenerative disorders, significantly impairing patient quality of life while escalating healthcare expenditures worldwide. In this article, we mainly reviewed the association between apelin and motor system degenerative diseases. As discussed above, apelin plays a pleiotropic role in the development of degenerative diseases of the motor system. The mainstream view is that in bone-related aspects, apelin can delay the progression of osteoporosis through different mechanisms and signaling pathways. Nevertheless, apelin promotes the pathophysiology of OA. When it comes to muscle-related aspects, apelin fights the progression of sarcopenia.

The above results are mainly based on animal experiments. However, further large-scale clinical trials are needed to perform for the benefit of determining whether apelin can be used as a reliable biomarker for degenerative diseases of the human motor system. As mentioned above, a few of animal experiments support the opposite result. What’s more, A small number of clinical trials have suggested that apelin may lack the association with motor system degenerative diseases [110, 111, 156], which makes it challenging to further prove whether apelin has reliable clinical significance in practical applications. However, there is more evidence to support that there is a close relationship between apelin and degenerative diseases of the motor system [15, 19]. When there are conflicting conclusions between basic research (such as cell and animal experiments) and clinical research, more rigorous epidemiological and translational medicine strategies are needed to validate and resolve these differences. Firstly, the internal validity of clinical research should be reassessed, potential selection bias or information bias should be identified, and subjects should be strictly screened to avoid confounding factors. Multi-center clinical cohort studies can also be conducted to further reduce bias. Due to the objective differences in physiology between humans and animals, as well as the influence of in vivo and in vitro experimental conditions on experimental results, and the existence of many unexplored physiological mechanisms in the medical field that may also affect experimental results, the results of cell and animal experiments cannot be fully extrapolated to clinical applications. It is crucial to adopt reverse transformation, within the framework of medical research ethics, by using humanized animal models to reproduce basic research results in a near clinical environment, while using techniques such as single-cell sequencing to reveal cellular heterogeneity that may be masked by population data.

Further research in the future can focus on the relevant signal transduction of apelin, to further elucidate the relationship between upstream pathophysiological stimuli and downstream signals. At the same time, attention should be paid to the heterogeneity of the target audience of apelin, and basic research should be conducted on different cells, tissues, and organs to provide reliable basis for the formulation of precise treatment strategies in the future. The specific biological mechanism of apelin regulation may be an interesting strategy for the prevention as well as treatment of degenerative diseases of the motor system, and may be a potential and promising therapeutic target, which should attract further attention in future research.
